# Relationship between Selected Functional Performance Parameters and the Occurrence of Anaemia in Hospitalized Females and Males Aged 80 and More

**DOI:** 10.3390/ijerph192013179

**Published:** 2022-10-13

**Authors:** Justyna Brożonowicz, Agnieszka Ćwirlej-Sozańska, Bernard Sozański, Ewa Orzech-Janusz, Anna Garus, Magdalena Grzesik, Anna Wilmowska-Pietruszyńska, Agnieszka Wiśniowska-Szurlej

**Affiliations:** 1 Institute of Health Sciences, College of Medical Sciences, University of Rzeszow, 35-310 Rzeszow, Poland; 2 Institute of Medicine, College of Medical Sciences, University of Rzeszow, 35-310 Rzeszow, Poland; 3 Geriatric Department at the District Hospital Named by Henryk Jankowski in Przeworsk, 37-200 Przeworsk, Poland; 4 Faculty of Medicine, Lazarski University, 02-662 Warsaw, Poland

**Keywords:** ageing, health, anaemia, handgrip strength, hospitalization, physical performance

## Abstract

Anaemia is considered a factor that significantly increases the risk of disability and mortality in the elderly. Among the hospitalized elderly, the incidence of anaemia is higher than in the general population, which necessitates extensive diagnostics for anaemia in this group. The aim was to assess the relationship between the occurrence of anaemia in hospitalized females and males, aged 80 years and more, and selected determinants of motor fitness. The analysis covered 91 females and 57 males aged 80 and more. The study implemented haemoglobin level, handgrip strength, a lower limb strength, mobility and balance measurement. The analysis used the logistic regression model and the cut-off point was determined by ROC curve. A 1 kg increase in muscle strength reduced the risk of anaemia in a group of males by 12%. The value of handgrip strength from which anaemia can be predicted in a group of males is 24.4 kg. In the female group, no statistically significant correlations were found. There is a need to continue research in this area with the participation of a larger group of respondents to look for potential factors that may be related to anaemia, in order to find non-invasive diagnostic tools useful for elderly people.

## 1. Introduction

According to data from the World Health Organization (WHO), in 2020 the global population of people over 60 years of age was 1 billion. According to statistical forecasts, by 2050 this number could double to 2.1 billion. It should be emphasized that the number of people in the oldest age groups is growing faster than the total number of elderly people. By 2050, that number could triple compared to 2020 to 426 million [1]. Statistical forecasts for the Polish society also indicate that over the years 2020–2050, the percentage of people in the oldest age groups (80 and over) will increase from 4.4% to 10.4%, that is, more than double. According to statistical forecasts, the percentage of people aged 65 and more in the structure of the Polish society will increase 1.7 times [2]. Moreover, the sex gap increases with age in society. In Poland, there are 129 females per 100 males in the 60–64 age group, and 208 females in the 80–84 age group [3].

An increase in the incidence of health problems among older people is also observed with age. Anaemia is one of the common health problems in older people that affect their functioning. According to the WHO, this disease is defined as a state of an insufficient number of red blood cells, which makes it impossible to meet the physiological needs of the body. The most commonly used biochemical parameter in diagnosing anaemia is the level of haemoglobin in the blood [4]. According to data obtained in a nationwide survey focusing on the health condition of the elderly (Pol Senior), the problem of anaemia in the elderly in Poland concerns 13.3% of females and 20.4% of males aged 80 years and more. In addition, an increasing sex diversity in the incidence of anaemia is observed above the age of 80. In people over 90 years of age, anaemia may affect up to 45.3% of males and 30.1% of females [5]. According to studies, the incidence of this problem also increases significantly in patients in hospital conditions and in institutional care centres. It is assumed that due to the dynamic aging of societies, the problem of anaemia will affect an increasing number of people in the oldest age groups [6,7].

The most common causes of anaemia in hospitalized males and females include the co-incidence of chronic diseases in which anaemia is secondary. In many cases, the causes of this condition in the elderly are also classified as “unexplained” [8]. An anaemia problem with an ambiguous cause may affect up to one-third of people over the age of 65, and this percentage increases with age [9]. Nillson-Ehle et al., while conducting the study among the Swedish population, did not find a clear cause of anaemia in 33% of individuals at the age of 70, 23% at the age of 75 and 36% at the age of 81 [10]. Due to the fact that the degree of anaemia in the elderly is mild in many cases, it is falsely perceived as a minor problem, especially for people with multiple morbidities. At the same time, an increasing number of scientific reports indicate serious consequences of anaemia in the elderly [11].

With reference to many clinical trials conducted thus far, anaemia has been shown to be a significant factor in the increased risk of mortality, more frequent cardiovascular events, cognitive impairment and disability among older people [12,13,14]. Joosen et al. indicated that severe anaemia while the patient is discharged from hospital is associated with an increased risk of death within a 12-month period [15]. Moreover, Maraldi et al. presented that lower haemoglobin levels in hospitalized older patients were associated with a lower likelihood of regaining independence in everyday activities after the end of a hospital stay [16]. According to Hirani et al., the incidence of anaemia in the elderly is also associated with weakness syndrome, and the results of the longitudinal studies suggest that the incidence of anaemia precedes the development of weakness syndrome in older males over several years of observation [17]. It has also been shown that the presence of even mild anaemia in the elderly is associated with an increased likelihood of falling and developing dementia [18,19,20,21]. According to Corona et al., the association of anaemia in elderly people with the occurrence of chronic diseases and decreased functional independence may be associated with decreased production of renal hormones, increased production of pro-inflammatory cytokines, which may lead to a decrease in the sensitivity to erythropoietin [22].

Despite the fact that currently measuring the concentration of haemoglobin in the blood is considered a standard in the diagnosis of anaemia, many authors are looking for non-invasive methods [23,24]. Simple, widely available and cheap tests are sought, which could be the basis for extending diagnostics to laboratory tests. Taking into account the assumptions of comprehensive geriatric care, simple diagnostic screening methods should be available not only to the physician, but also to other members of the interdisciplinary team (nurses/physiotherapists) [25]. Therefore, the relationship between the occurrence of anaemia and anthropometric variables or functional performance parameters is being sought more often.

Most of the studies carried out so far to investigate factors associated with anaemia have been conducted in the group of older people aged 65 years and older. Studies on the relationship between anaemia and the level of disability, sociodemographic factors and results obtained in functional tests in people in the oldest age groups were performed by a few researchers [21,26]. However, according to the literature review, none of research was conducted in the group of patients hospitalized in Poland. It is worth mentioning that Den Elzen et al., based on their studies analysing the relationship between anaemia, mortality and functional fitness of people over 85 years of age found that the factors determining the health and fitness of people in the oldest age groups differ from “younger older” [26].

The aim of the study was to assess the relationship between the occurrence of anaemia in a group of hospitalized females and males aged 80 years and more and selected determinants of motor fitness in order to predict the incidence of anaemia in the studied groups. The following research questions were asked in the study (RQ):

1. Is there a relationship between the occurrence of anaemia and selected motor fitness factors in hospitalized elderly people aged 80 and more?
2. Can the values of individual parameters of motor fitness constitute a predictive marker for the diagnosis of anaemia in hospitalized elderly people aged 80 and more?

## 2. Materials and Methods

### 2.1. Study Group

This cross-sectional study was conducted among patients hospitalized in the Geriatric Ward of the Hospital in Przeworsk. The hospital in Przeworsk is one of the three hospitals in the Podkarpackie Voivodeship (in south-eastern Poland) that were invited to participate in the study. At the same time, it is the only hospital that has expressed a willingness to cooperate in research. The study was conducted from January to September 2019. The duration of the study was determined on the basis of the designated sample size and statistical data on the average rate of hospitalization in geriatric wards of people over 80 in the studied region [27,28].

The inclusion criteria in the study were the age of the subjects at least 80 years, no cognitive impairment (AMTS Abbreviated Mental Test Score >6 points) and the patient’s functional condition enabling the researchers to perform tests for mobility and balance (ADL ≥ 3). The exclusion criteria were the presence of serious systemic diseases leading to complete inability to perform functional tests as well as the occurrence of infections, acute inflammation, diseases involving acute or chronic bleeding, haematological cancer or congenital hemoglobinopathies, as well as transfusion of red blood cells within 7 days before starting the test. Moreover, the exclusion criteria also included the presence of serious visual impairment that prevented the patient from performing functional tests and/or hearing impairment that prevented the patient from hearing and understanding the commands and questions asked by the physiotherapist and provide a reliable answer.

The study was approved by the Bioethics Committee of the University of Rzeszów (Resolution No. 2/12/2018). In accordance with the assumptions of the Helsinki Declaration, all subjects were informed about the purpose and procedure of the study, and they were asked to give informed consent to participate in the study. Lack of informed consent to participate in the study was an additional exclusion criterion for the study.

### 2.2. Study Procedure

The study was carried out within 48 h after the admission to the Geriatric Ward. The study was conducted in four stages, and all research procedures were performed in the morning. There was an hour break intended for rest for the patient between the individual stages of the study. The entire research procedure was coordinated by the attending physician, who qualified the patient in the first stage to participate in the study, on the basis of the adopted inclusion and exclusion criteria. The second part of the study involved taking blood samples and sending them for laboratory analysis. The third stage was based on conducting a direct interview by a trained member of the research team, which was used to obtain basic sociodemographic data of the subjects. The fourth stage of the study was a subjective examination carried out by a physiotherapist in order to assess the functional state of the subjects by means of selected tests. However, information on the state of health was obtained based on the analysis of patient’s medical records.

### 2.3. Variables

#### 2.3.1. Dependent Variable

##### The Occurrence of Anaemia

Anaemia was diagnosed based on the level of haemoglobin in the blood. Results below 12 g/dL in females and below 13 g/dL in males indicated anaemia, which is consistent with WHO guidelines [4].

The measurement of haemoglobin in the blood was carried out in the morning, keeping a regime of a 12 h period without food intake. Blood samples were collected by a nurse working in the ward, following hospital procedures implemented while collecting samples for microbiological tests. The haemoglobin level was assessed using the cyanmethemoglobin method in the laboratory of the hospital in which the study was conducted [29].

#### 2.3.2. Independent Variables

##### Functional State Assessment

*Handgrip strength*

Each patient was assessed for upper-limb strength by means of the handgrip strength measurement of the dominant hand. An electronic hand dynamometer (JAMAR PLUS + Digital Hand Dynamometer, Patterson Medical) was used for the study. The mean result of the three trials was assumed as the final result [30].

*Lower-limb strength*

In order to assess the lower-limb strength, a 30 s chair stand test was performed. The final result for this test was the number of repetitions of sits–stands cycle a person could complete in 30 s [31].

*Mobility*

The Timed Up&Go test was used to assess gait, mobility and risk of falling, in which the time was measured in seconds while the person needed to stand up from the chair, walk a distance of 3 m, turn back, walk back a distance of 3 m and return to a sitting position on the chair with the back resting on the backrest [32].

*Balance*

Balance was assessed using the Berg Balance Scale. The test allowed the researchers to assess static and dynamic balance while assessing a patient in 14 positions, and the maximum result to be obtained was 56 points [33].

*Confounding variables*

Basic sociodemographic data were collected and some medical information was based on the analysis of patient’s medical records. The collected data concerned age (confounding variable), education level (the responses were classified according to the following categories: 0–7 years and above 7 years), place of residence (town/village), marital status (living with a spouse or partner/widow/widower or lonely) and type of work performed in the past (manual workers/non-manual workers). Researchers collected information on the number of comorbid chronic diseases and the number of taken medications by individuals. The medications taken by the subjects chronically as well as medications ordered during admission to the geriatric ward were taken into account. Each person was also measured for height and weight and based on the obtained data, and then the Body Mass Index (BMI) kg/m^2^ was determined.

### 2.4. Statistical Analysis

Statistica 13.1 software was used to perform the data analysis. Basic sociodemographic data and the distribution of results obtained in functional tests were presented by the use of descriptive statistics. The analysis employed the following tests: Chi-square tests (for qualitative variables) and the Mann–Whitney U test (for quantitative variables), as well as Fisher and Student’s *t*-tests for two independent samples. In order to identify potential factors significantly associated with anaemia, a binary logistic regression model was used (unadjusted and adjusted for age), which allowed the researchers to estimate the odds ratio (OR) with their 95% confidence interval (95% CI). The level of statistical significance in the analysis was assumed at *p* < 0.05.

To evaluate the diagnostic power of the functional performance tests that were statistically significantly related to anaemia in a binary logistic regression model, the ROC (Receiver Operating Characteristic) curve was determined and the area under the curve (AUC) was assessed. Additionally, the best cut-off points were determined in order to find the values of functional tests that may be prognostic values for the occurrence of anaemia. The optimal cut-off values were obtained from the maximal Youden’s Index, and the best combination of sensitivity and specificity.

## 3. Results

**Figure 1:**
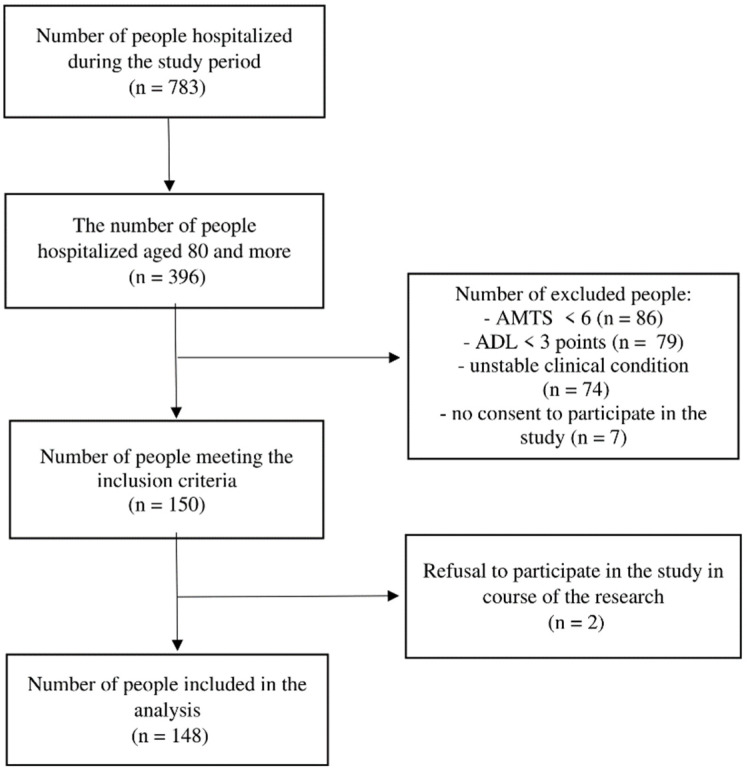
Flow diagram presenting the stages of including subjects in the analysis.

In the course of the study, 783 people were hospitalized in the ward, including 396 aged 80 and more. After taking into account the exclusion criteria concerning health and functional status of the subjects, 246 people were excluded from further study. In addition, of those who met the inclusion criteria, 2 people left the study after it had started. After considering all inclusion and exclusion criteria, 148 patients were included in the final analysis (Figure 1).

The final analysis covered a group of 91 females and 57 males aged 80–96. The average age in the studied group was 84.9 years, while the average BMI level was 28.1 kg/m^2^. The vast majority of the group were people whose education level was 0–7 years (75.0%). Most (77.0%) of the respondents lived in the village. Taking into account the type of work done in the past, 90.5% were manual workers. Most of the respondents were living without a partner (widow/widower or lonely)—67.6%. The subjects included in the study took an average of 8.1 drugs and suffered from an average of 5.6 chronic diseases.

Anaemia in the study group was diagnosed in 85 examined people (including 56.0% of females and 57.9% of males). It was found that males with anaemia were statistically significantly older than males without anaemia (*p* = 0.004). Males with anaemia lived more often in the countryside (*p* = 0.035) and had a low level of education amounting 0–7 years (*p* = 0.039). In the female group, there were no statistically significant correlations between the incidence of anaemia and the analyzed sociodemographic factors (Table 1).

Males with anaemia were characterized by statistically significant lower mean results in the handgrip strength range—19.2 kg in the group of males with anaemia and 26.3 kg in the group of males without anaemia (*p* < 0.001), as well as lower mean results in the field of lower-limb strength—5.2 repetitions in the 30 s chair stand test in males with anaemia and 8.8 repetitions in males without anaemia (*p* = 0.001). It was also indicated that males diagnosed with anaemia were characterized by statistically significant lower mean results obtained on the Berg Balance Scale—30.5 points in males with anaemia and 42.6 points in males without anaemia (*p* < 0.001), and higher mean results obtained in the Timed Up & Go test, indicating worse mobility (*p* < 0.001). With reference to the group of females with and without anaemia, there were no statistically significant differences between the mean scores obtained in the analyzed functional tests (Table 2).

Due to the fact that the analyzed indicators of functional performance were statistically significantly different only in the group of males with and without anaemia, further analysis in the form of a logistic regression model was carried out only for the male sex. The model that was used to assess factors associated with the incidence of anaemia in the male group was well fitted to the data, which is confirmed by the results of the Hosmer–Lemeshow test (HL = 6.428, *p* = 0.599). Using a logistic regression model, a statistically significant effect of handgrip strength in males on anaemia occurrence was demonstrated (OR = 0.87, 95% Cl = 0.78–0.98, *p* = 0.022). After adjusting for age, a significant association was also found between handgrip strength and the occurrence of anaemia (OR = 0.88, 95% Cl = 0.79–1.00, *p* = 0.041)—with an increase of 1 kg in male’s handgrip strength reducing the risk of anaemia in this group by 12% (Table 3).

**Figure 2:**
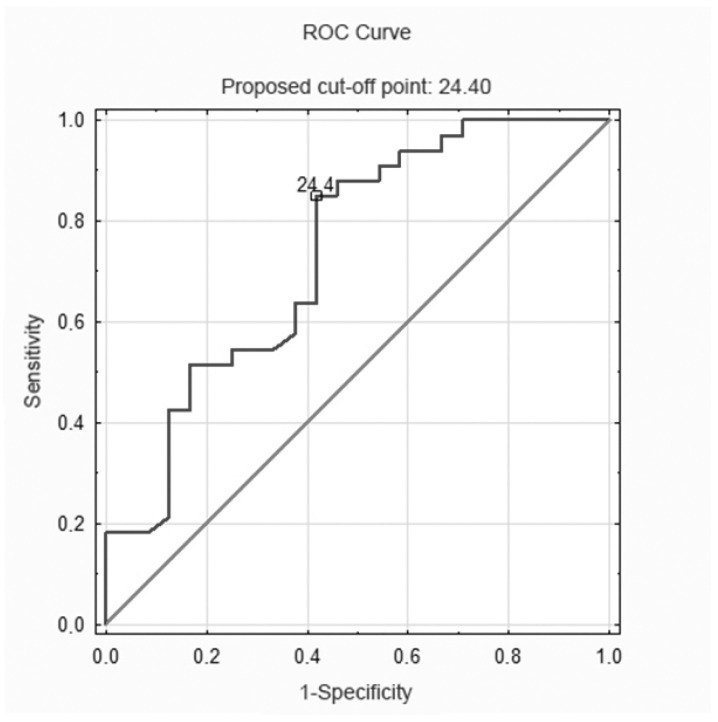
ROC curve parameters for dominant upper-limb strength, which are predictors of anaemia in the male group, AUC: 0.736 (0.602–0.870), cut-off point: 24.4 kg.

Due to the fact that the logistic regression model showed a significant statistical influence in only one of the analyzed functional tests (handgrip strength) on the occurrence of anaemia in the group of males, to determine the value of handgrip strength, from which anaemia could be predicted in this group, the ROC curve was determined. The area under the curve (AUC) was higher than 70%, which means that the value of handgrip strength can be considered as a parameter that differentiates the incidence of anaemia in males over 80 years of age. It was shown that the cut-off point for anaemia in the study group was 24.4 kg, with a sensitivity of approximately 85% (Figure 2).

## 4. Discussion

The study presented statistically significant differences in terms of handgrip strength, lower-limb strength, balance and mobility in males with and without anaemia. In a logistic regression model adjusted for age it was found that an increase in handgrip strength by 1 kg in the group of males reduced the risk of anaemia in this group by 12%. In addition, by means of the ROC curve parameters, it was determined that the handgrip strength value amounting 24.4 kg was the point below which anaemia could be predicted in a group of males.

The percentage of people diagnosed with anaemia in the conducted study was over 57% and it was close to the results reported by Tay et al. who indicated that in the group of hospitalized people aged 85 and more, anaemia affected 59% of respondents [34]. Similar results were also achieved by Italian researchers who recorded anaemia in 58% of hospitalized people aged 80 and over [35].

With regard to our study, the incidence of anaemia was similar in both females and males. Gaskell et al., based on the literature review, which included 28 publications indicating the relationship between the incidence of anaemia in the elderly by sex, demonstrated only slight differences in the incidence of anaemia in females and males [36]. Moreover, Al Obaidely et al. stated in their research that anaemia occurred in females more often below the age of 80, whereas it was recorded more often in males who were over 80 years old [37]. According to Ferruci et al., the occurrence of anaemia in the elderly is closely related to testosterone levels, owing to a severe decrease in testosterone levels in the blood, which causes the intensification of metabolic processes in the bone marrow, and, consequently, slowing erythropoiesis and increasing the likelihood of anaemia, especially in males [38]. The results of some studies indicate that testosterone deficiency may occur in up to 50% of males over the age of 80, which may explain the reports of some authors that anaemia is more common in this group [39]. According to the results of Lewerin et al., hemoglobin levels below normal in older males may also be associated with the decrease in estradiol levels observed with age, which is responsible for iron uptake in liver cells [40].

Our own study revealed that handgrip strength was associated with the incidence of anaemia in males, with no significant relationship for this parameter in the group of females. The results obtained in the research in respect to the relationship between the incidence of anaemia and individual determinants of functional performance were similar to those obtained by Gi et al. The authors noted that handgrip strength was more closely associated with the incidence of anaemia in the group of males than in the group of females [41]. Similar results were also received by Hirani et al. who showed a relationship between haemoglobin levels and sarcopenia in older males. The authors revealed in their studies that mild and moderate anaemia, as well as haemoglobin levels below 14.2 g/dL were associated with the occurrence of sarcopenia in a group of males, defined e.g., as a decrease in muscle strength below 26 kg. In addition, the authors pointed out that an increase in haemoglobin by 1 g/dL was significantly associated with a reduced risk of dropping gait speed, a decrease in handgrip strength and lower-limb strength, as well as a decrease in the risk of sarcopenia in the studied group of males [17]. Shmiztu et al. observed in their study the relationship between the occurrence of anaemia and low muscular strength in males, especially among males characterized by an additionally high rate of hepatocyte growth [42]. On the other hand, different results to our own work were obtained by Santos et al. The authors showed that the occurrence of anaemia in a group of females over 65 year of age was associated with a lower grip strength, and the value from which anaemia can be predicted in this group is 18 kg [43]. Similarly, Payne et al., who researched people living in rural areas of southern Africa, demonstrated that muscle strength was an independent factor of anaemia incidence only in the group of females. However, it should be emphasized, that there are significant age, cultural and economic differences in relation to the study [44].

Taking everything into account, on the grounds of our research and previous literature reports, it is observed that muscle strength is a factor strongly associated with anaemia among people in the oldest age groups [21,45,46,47]. The conducted literature review allows for the assumption that the relationship between the occurrence of anaemia and handgrip strength may be associated with impaired capacity for carrying oxygen, resulting in local hypoxia of the muscles, and consequently a weakening of their strength and deterioration of performance [17]. In addition, fatigue associated with anaemia is considered an important factor associated with limitations in taking physical activity and engaging in everyday activities. This additionally contributes to the decrease in muscle mass and strength resulting from inactivity [47,48]. Many of the studies conducted thus far have indicated a positive effect of resistance exercises on improving muscle mass and strength in the elderly. Some reports also suggest a relation between resistance training and stimulation of erythropoiesis, which increases the mass of haemoglobin and red blood cells and improves the ability to carry oxygen [49]. However, further research is needed to assess the safety of this form of exercise in people in the oldest age groups and in different degrees of clinical anaemia [17,49].

The performed research has both strengths and weaknesses. The strength of the study is conducting the survey with patients in the oldest age groups in hospital. To our knowledge, the study is the first to assess the diagnostic value of selected functional tests in predicting the incidence of anaemia in females and males in the oldest age groups hospitalized in Poland.

There are several limitations with this research. One of the limitations with reference to the study may be exclusion criteria adopted in the study, excluding people with significant cognitive impairment and limitations in ADL with significant mobility impairment. Therefore, the adopted assumptions could have resulted in the exclusion of a significant number of people with anaemia and, consequently, a reduction in the actual proportion of people with anaemia in the study population. Secondly, the study was conducted on a small population of elderly people, which means that the obtained results should be interpreted with caution in relation to the general population. Due to the cross-sectional design of the study, caution should be also exercised in interpreting the causal relationship between the analyzed variables.

## 5. Conclusions

The results obtained in the study have indicated a significant relationship between handgrip strength and the incidence of anaemia in hospitalized males aged 80 years and more. It was shown that the handgrip strength value, from which the occurrence of anaemia can be predicted in the group of males, is 24.4 kg. In the group of females, no relationship was found between the occurrence of anaemia and the analyzed functional performance parameters.

There is a need to continue research in this area with the participation of a larger group of respondents to look for potential factors that may be related to anaemia, in order to find non-invasive diagnostic tools useful for elderly people.

## Figures and Tables

**Table 1:** Characteristics of the group depending on the incidence of anaemia in the group of females and males (n = 148).

Variable	Total Group	Anaemia in Females		*p*-Value (F)	Anaemia in Males		*p*-Value (M)
		Yes	No		Yes	No	
**Sociodemographic variables**							
Age (average, SD)	84.9, 3.4	84.3, 3.2	85.3, 3.0	0.060 ^†^	86.5, 4.0	83.5, 2.5	0.004 ^†,^*
Education level (n,%)							
0–7	111, 75.0	38, 74.5	33, 82.5	0.448 ^§^	27, 81.8	13, 54.2	0.039 ^§,^*
>7	37, 25.0	13, 25.5	7, 17.5		6, 18.2	11, 45.8	
Place of residence (n,%)							
Town	34, 23.0	12, 23.5	7, 17.5	0.606 ^§^	5, 15.2	10, 41.7	0.035 ^§,^*
Village	114, 77.0	39, 76.5	33, 82.5		28, 84.8	14, 58.3	
Marital status (n,%)							
Living with a spouse or partner	48, 32.4	7, 13.7	9, 22.5	0.406 ^§^	20, 60.6	12, 50.0	0.426 ^‡^
Widow/widower or lonely	100, 67.6	44, 86.3	31, 77.5		13, 39.4	12, 50.0	
Type of work performed in the past (n,%)							
Manual workers	134, 90.5	47, 92.1	35, 87.5	0.499 ^§^	30, 90.9	22, 91.7	1.000 ^§^
Non-manual workers	14, 9.5	4, 7.9	5, 12.5		3, 9.1	2, 8.3	
**Health status variables**							
Incidence of anaemia (n,%)	85, 57.4	51, 56.0	40, 44.0	-	33, 57.9	24, 42.1	0.757 (F/M)
BMI kg/m^2^ (mean, SD)	28.1, 5.8	29.3, 6.0	29.36, 6.2	0.987 ^†^	25.3, 4.1	27.0, 5.3	0.210 ^†^
Number of medications taken (mean, SD)	8.1, 3.0	8.4, 2.7	8.18, 2.6	0.700 ^¶^	7.9, 3.5	6.9, 3.4	0.071 ^¶^
Number of comorbid chronic diseases (mean, SD)	5.6, 3.0	5.9, 2.7	6.20, 3.7	0.981 ^†^	5.1, 2.8	4.8, 2.6	0.936 ^†^

*—statistical significance; SD—Standard Deviation; BMI—Body Mass Index, F—female; M—male, ^†^ Mann–Whitney U test, ^‡^ Chi-square Pearson’s test. ^§^ Fisher’s test. ^¶^ Student’s *t*-test.

**Table 2:** Functional performance indicators for anaemia in females and males (n = 148).

Variable	Total Group (Mean, SD)	Anaemia Incidence		*p*-Value
Females:		Yes (Mean, SD)	No (Mean, SD)	
Handgrip strength	14.0, 4.9	13.4, 4.9	14.7, 4.8	0.207 ^†^
Lower-limb strength (30 s chair stand)	5.9, 3.2	5.6, 3.3	6.3, 3.2	0.317 ^†^
Balance (Berg Balance Scale)	34.2, 12.3	34.1, 13.0	34.3, 10.2	0.795 ^‡^
Mobility (Timed Up & Go)	29.6, 22.0	31.0, 24.1	28.5, 19.2	0.879 ^‡^
**Males:**				
Handgrip strength	22.2, 7.7	19.2, 6.4	26.3, 7.6	<0.001 ^†,^*
Lower-limb strength (30 s chair stand)	6.7, 3.9	5.2, 3.3	8.8, 3.8	0.001 ^‡,^*
Balance (Berg Balance Scale)	35.6, 13.2	30.5, 13.4	42.6, 9.0	<0.001 ^‡,^*
Mobility (Timed Up&Go)	29.5, 29.1	38.6, 35.4	16.9, 5.6	<0.001 ^‡,^*

*—statistical significance; SD—Standard Deviation. ^†^ Student’s *t*-test. ^‡^ Mann-Whitney U test.

**Table 3:** Logistic regression model unadjusted and adjusted for age assessing the odds ratio for the incidence of anaemia in a group of males.

		Unadjusted				Adjusted for Age		
Variable	Odds Ratio (OR)	Confidence Interval (Cl 95%)	Confidence Interval (Cl 95%)	*p*-Value	Odds Ratio (OR)	Confidence Interval (Cl 95%)	Confidence Interval (Cl 95%)	*p*-Value
Handgrip strength	0.87	0.78	0.98	0.022 *	0.88	0.79	1.00	0.041 *
Lower-limb strength (30 s chair stand)	1.07	0.78	1.47	0.665	1.06	0.78	1.49	0.664
Balance (Berg Balance Scale)	0.94	0.85	1.05	0.294	0.96	0.86	1.08	0.525
Mobility (Timed Up&Go)	1.11	0.96	1.27	0.151	1.09	0.96	1.25	0.186

*—statistical significance.

## Data Availability

The datasets used and analyzed in the current study are available from the corresponding author on reasonable request.
